# Electrochemical Synthesis of Novel 1,3-Indandione Derivatives and Evaluation of Their Antiplatelet Aggregation Activities 

**Published:** 2013

**Authors:** Salimeh Amidi, Farzad Kobarfard, Abdolmajid Bayandori Moghaddam, Kimia Tabib, Zohreh Soleymani

**Affiliations:** a *Department of Medicinal Chemistry, School of Pharmacy, Shahid Beheshti University of Medical Sciences, Tehran, Iran.*; b *Phytochemistry Research Center, Shahid Beheshti University of Medical Sciences, Tehran, Iran.*; c *Department of Engineering Science, College of Engineering, University of Tehran, P.O. Box 11155-4563, Tehran, Iran.*

**Keywords:** Electrochemical synthesis, 1,3-Indandione, Platelet aggregation inhibition

## Abstract

Electrochemical oxidation of some selected catechol derivatives, using cyclic voltammetry, in the presence of different 2-aryl-1,3-indandiones as nucleophiles, resulted in electrochemical synthesis of new 1,3- indandione derivatives in an undivided cell in good yield and purity. A Michael addition mechanism was proposed for the formation of the analogs based on the reaction conditions which were provided in electrochemical cell. The *in-vitro *antiplatelet and anticoagulant activity of these compounds was evaluated, using arachidonic acid (AA) and adenosine diphosphate (ADP) as the platelet aggregation inducers. The results show that the incorporation of catechol ring in 1,3-indandione nucleus leads to the emergence of antiplatelet aggregation activity in these compounds. The compounds may exert their antiaggregation activity by interfering with the arachidonic acid pathway.

## Introduction

Stroke and cardiovascular diseases are the major reasons of morbidity and mortality in the world (21.9% of total death) which are mainly caused by thrombus formation and platelet activation (1). 

Antiplatelet agents such as aspirin (acetylsalicylic acid), clopidogrel or ticlopidine and anticoagulants such as warfarin are currently two predominant groups of orally consumable drugs in standard therapeutic protocols for prophylaxis and treatment of venous thrombosis and reducing the risk of recurrent myocardial infarction (2-3). Combination therapy with anticoagulants and platelet aggregation inhibitors is frequently used in high risk patients in order to achieve more efficient clinical results (4-5). However, these medications exhibit some side effects such as aspirin-related gastrointestinal ulcers, allergy and bleeding or warfarin-induced skin necrosis (3, 6). Furthermore, the efficacy of these drugs is not still satisfactory. Therefore, medicinal chemists are still trying to find new drug candidates in this therapeutic area (7). 

**Figure 1:**
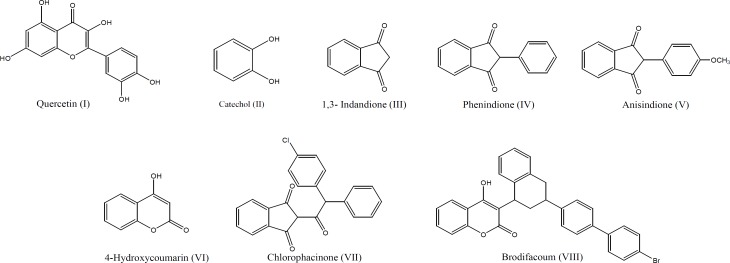
Chemical structures of quercetin, catechol, 1,3-Indandione and coumarin derivatives

Quercetin (I) is a naturally occurring flavonoid structurally related to coumarins with a catechol ring at its position 2 of coumarin scaffold (Figure 1). It was also shown to be effective inhibitor of platelets aggregation in dogs and monkeys (8). 

**Figure 2:**
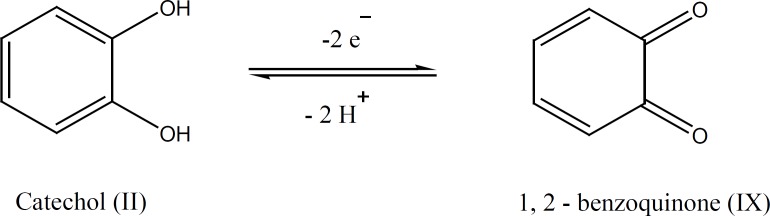
Electrochemical conversion of catechol to 1,2-benzoquinone

Catechol (II) is a diphenolic derivative which could be electrochemically converted to 1,2-benzoquinone by a reversible two-electron, two-proton oxidation (Figure 2). 

The electrochemically generated *o*-benzoquinones are quite reactive intermediates which in a proper condition can be attacked by a variety of nucleophiles and undergo various reactions such as Michael addition.

Electrochemical methods are considered as green methods for the synthesis of organic compounds and our goal in the present study was to use these methods for preparation of a few novel catechol containing structures with potential antiplatelet aggregation activity.

1,3-Indandiones (III) were selected as the compounds with nucleophilic carbon. 1,3-Indandione is an aromatic *β*-diketone. In the solid state it occurs as a diketone while in solution, it is partially enolized and the enolate anion exhibits significant delocalization and the highest electron density is on the second carbon.

If catechol is oxidized to *o*-benzoquinone in the presence of indandione derivatives, a Michael addition reaction will occur between indandione and *o*-benzoquinone resulting to the formation of 2-substituted indandione.

1,3-Indandione derivatives such as phenindione (IV) and anisindione (V) are a group of anticoagulants with anti vitamin K activity similar to 4-hydroxycoumarins (VI) (Figure 1).

Catechols are known to have antiplatelet aggregation activity due to their prominent redox power. Kitagawa *et al*, (9) have reported the inhibitory effect of polyhydric phenols against arachidonic acid-induced aggregation of rabbit platelets and they found catechol as the most potent non-substituted dihydric phenols.

In fact, it has been reported that various phenolic compounds such as quercetin inhibit platelet cyclooxygenase (10).

**Figure 3:**
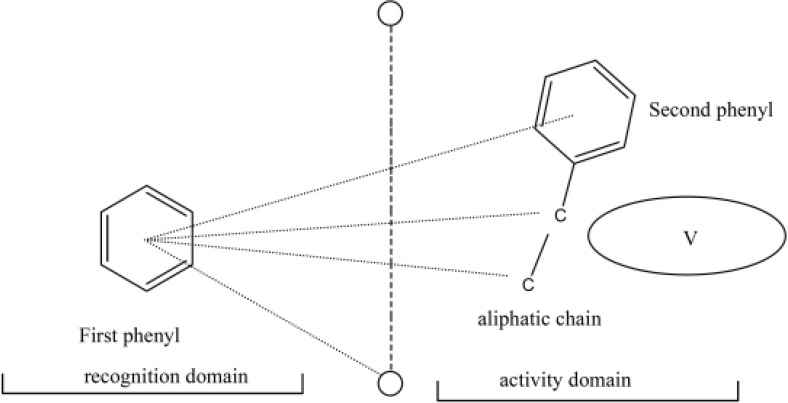
Main parameters of the biophore model. V is excluded volume (11).

Dolmella *et al. *(11) have proposed a three-dimensional (3D) biophore model that explains the requirements for efficient indandione and coumarin derivatives. They have suggested two domains in the structure of these derivatives: a recognition domain which consists of a phenyl ring plus two negatively charged oxygens (recognition flag), and an activity domain which starts half way between the two oxygens on a short aliphatic chain and consists of a second phenyl ring attached to the terminal atom of the chain (Figure 3).

 Indandione and coumarin derivatives, with a bulky lipophilic side chain at activity domain such as chlorophacinone (VII) and brodifacoum (VIII), enjoy more potency due to establishing an additional drug-receptor interaction by this extra anchoring group (Figure 1) (11).

Our goal in this study was incorporating a catechol ring to the structure of indandione derivatives in the activity domain of Dolmella’s model. The catechol ring is assumed to increase the resemblance of these compounds to quercetin and they are thus expected to show antiplatelet aggregation activity.

Different methods have been used for the synthesis of 1,3-indandione derivatives with substitution at position 2. Beringer *et al*. (12) and Matano *et al*. (13) reported phenylation of 1,3-Indandione with diaryliodonium salts and *α*-alkenylation of *β*-dicarbonyl compounds with alkenyltriarylbismuthonium salts, respectively. The Friedel-Crafts methods were also reported for the derivatization of 1,3-indandione at position 2 (14). In addition to these conventional methods, the electrochemical synthesis has also been used for preparation of indandione derivatives with catechol or 2,3-dimethylhydroquinone ring on their position 2 (15-17). The main advantages of the electrochemical methods lie in their high purity of the products, easy process and better control over the reaction process. They are also considered as green chemical synthesis methods (16-17).

Here, we used a simple one-pot electrochemical synthesis for preparing the proposed indandione derivatives and the antiplatelet aggregation activities of these new compounds were evaluated.

According to the previously reported methods for the synthesis of compound 15a-b (15-16), we described here a facile one pot electrochemical method for the synthesis of new indandione derivatives (16a-b, 17a-b, 18a-b, 19a-b).

**Figure 4:**
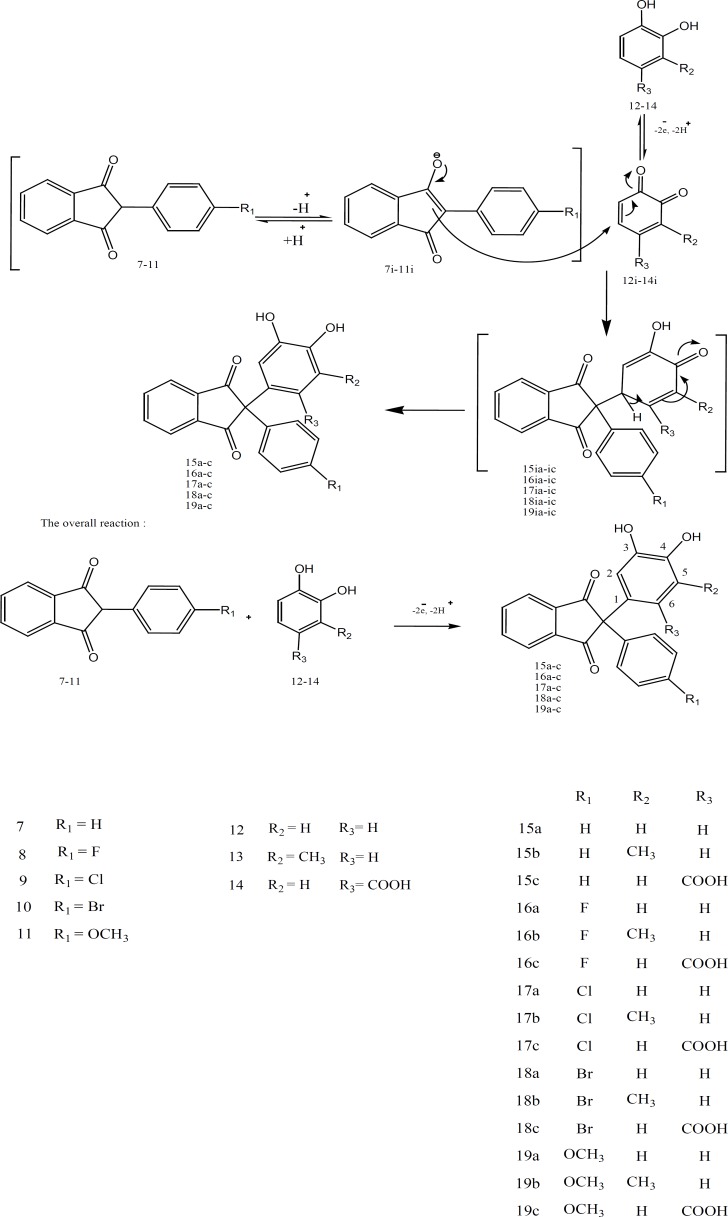
Mechanism of Michael addition reaction occurred in electrochemical cell

The key reactions involved the electrooxidation of catechol derivatives (compouds12-14) in the presence of indandiones (7-11) as nucleophiles. Compounds 7-11 are 1,3-diketones with an acidic methylene group at position 2, which is easily converted to their corresponding enolate ions (7i **– **11i). These can act as nucleophile in Michael addition reaction and add to the beta carbon of an *α,β*-unsaturated system. Compounds 12-14 could be electrochemically oxidized to its corresponding quinonic form (12i-14i). When the oxidation process takes place in the presence of 7-11, the methylene group of compounds 7-11 can attack the quinonic ring in a Michael type addition reaction. The quinine ring could then be reduced electrochemically, using a cyclic voltammetric process, to regenerate the reduced form of catechol ring (Figure 4).

**Figure 5:**
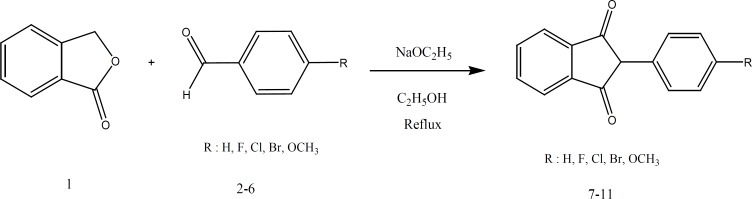
Synthesis route of 1,3-indandiones 7-11

Compounds 7-11 were prepared by condensation of phthalide (1) with appropriate benzaldehydes (2-6), in the presence of sodium ethoxide in one step (Figure 5) (18). 

The antiplatelet activity of these novel indandione derivatives (15a-b, 16a-b, 17a-b, 18a-b, 19a-b) was evaluated at 500 μM concentration by determining their ability to inhibit platelet aggregation induced by ADP (5 μM) and arachidonic acid (1.35 mM) according to Born method (19). 

## Experimental

**A**ll chemicals were reagent grade material and phosphate salts were of pro-analysis grade from Merck. 3-Methylcatechol and 4-methylcatechol were reagent grade materials from Aldrich. Cyclic voltammetry were performed, using Metrohm computerized voltammetric analyzer model 746 VA Trace Analyzer/747 VA stand. Controlled-potential coulometry and preparative electrolysis were performed using BHP2050 potentiostat/ galvanostat. The working electrode used in the voltammetry studies was a glassy carbon disc (1.8 mm diameter). The potential were measured versus the Ag/AgCl/KCl (3M) as a reference electrode and platinum wire was used as the counter electrode. In macroscale electrolysis, four carbon rods (8 mm diameter and 5 cm length) were used as working electrodes. The infrared (IR) spectra were recorded on Perkin Elmer IR spectrophotometer as potassium bromide discs. The proton nuclear magnetic resonance (^1^H-NMR) spectra were recorded on a 500 MHz Bruker spectrometer and the chemical shifts were expressed in *δ *(ppm) using TMS (tetramethylsilane) as an internal standard. The ^13^C-NMR spectra were recorded on a 250 MHz Bruker DPX_300_ spectrometer. The electrospray mass (ESI-MS) spectra were performed on Agilent 4610 triple quadrupole mass spectrometer. The melting points of the products were obtained on 9100 Electrothermal melting point apparatus. All the compounds were analyzed for C, H and N on a Costech model 4010 and agreed with the proposed structures within ± 0.4% of the theoretical values. ADP and AA which were used for platelet aggregation studies were purchased from Bio/Data, Corp. 

*General procedure for preparing derivatives 7-11*

2-(Aryl)-1,3 indandiones were prepared by the procedure previously reported (18). The synthesis of 2-(4-bromophenyl)-1,3- indandione (compound 10) is reported as an example. A mixture of phthalide (compound 1) (5.6 g, 0.04 mol) and aldehyde 5 (7.4 g, 0.04 mol) was added to a solution of sodium ethoxide (3.06 g, 0.045 mol) in absolute ethanol (40 mL) and refluxed for 1 h. Alcohol was removed and water (40 mL) was added. The residue was diluted with ice water (200 mL) and washed with ether (2× 40 mL). After acidifying with hydrochloric acid (6 M), the product was extracted into ether (40 mL), and then re-extracted with aqueous sodium bicarbonate, which precipitated upon addition of hydrochloric acid solution (6 M). The product was separated, dried, and recrystallized from methanol.

*2-(phenyl)-1, 3-indandione (7)*

Yield: 30%, mp: 148-151°C. *Anal. *Calcd for C_15_H_10_O_2_ : C, 81.07; H, 4.54. Found: C, 80.9; H, 4.53.

*2-(4-fluorophenyl)-1, 3-indandione (8)*

Yield: 32%, mp: 116-118 °C. *Anal. *Calcd for C_15_H_9_FO_2_: C, 75.00; H, 3.78. Found: C, 75.25; H, 3.76.

*2-(4-chlorophenyl)-1, 3-indandione (9)*

Yield: 20%, mp: 142-145 °C. *Anal. *Calcd for C_15_H_9_ClO_2_: C, 70.19; H, 3.53. Found: C, 70.29; H, 3.52.

*2-(4-bromophenyl)-1, 3-indandione (10)*

Yield: 26%, mp: 142-146 °C. *Anal. *Calcd for C_15_H_9_BrO_2_: C, 59.83; H, 3.01. Found: C, 59.9; H, 3.01.

*2-(4-methoxyphenyl)-1, 3-indandione (11)*

Yield: 23%, mp: 152-153 °C. *Anal. *Calcd for C_16_H_12_O_3_: C, 76.18; H, 4.79. Found: C, 76.2; H, 4.79.

*General procedures for electroorganic synthesis of 15a-c, 16a-c, 17a-c, 18a-c and 19a-c*

The mixture of water-acetonitrile (80:20), containing phosphate buffer (pH = 7.0 c = 0.2 M), was pre-electrolyzed at 0.3 V for catechol 12, 0.4 V for 3-methylcatechol 13 and 0.6 V for 3,4 dihydroxybenzoic acid 14 mixture. 1 mmole of 7-11 and 1 mmole of 12-14 were added to the cell with 4 graphite rods as working electrodes and Pt electrode as counter electrode. The potentials of working electrode were measured versus the Ag/AgCl/KCl as a reference electrode. The electrolysis was interrupted many times, when the current reached to 5% of the starting value, to wash the anodic electrode with acetone to reactivate it. The precipitated products were filtered off and washed with water/acetone mixture.

*2-(3,4-dihydroxyphenyl)-2-phenyl-2H-indene-1,3-dione (15a)*

Yield: 43%, mp: 198-200 °C. IR (KBr) cm^-1^: 3316, 1689 (C=O), 1525, 1429, 1253, 789. ^1^H-NMR (DMSO-d_6_, *δ*, ppm) : 6.42 (1H; dd, J = 8.28 Hz, J = 2.32 Hz; H-6 of catechol ring); 6.61 (1H; d, J = 2.32 Hz; H-2 of catechol ring); 6.68 (1H; d, J = 8.28 Hz; H-5 of catechol ring); 7.12 ( 2H, m, H-2 and H-6 of phenyl ring); 7.32 (3H, m, H-3, H-4 and H-5 of phenyl ring); 8.1 (4H, m, Hs of indandione ring); 9.03 (1H, s, OH); 9.06 (1H, s, OH). ^13^C-NMR (DMSO, δ, ppm): 67.2 (C-2 of indandione ring), 116.0, 116.3, 119.7, 124.5, 128.0, 128.4, 129.0, 129.5, 137.6, 138.7, 141.2, 145.7, 145.8, 200 (C=O). ESI-MS: Observed (M+H)^+^ = 331, (M+Na)^+^ = 353 (Calcd for C_21_H_14_O_4_ = 330.33). *Anal*. Calcd for C_21_H_14_O_4_ : C, 76.35; H, 4.27. Found: C, 76.30; H, 4.27.

*2-(3,4-dihydroxy-5-methylphenyl)-2-phenyl-2H-indene-1,3-dione (15b)*

Yield: 41%, mp: 215-217°C. IR (KBr) cm^-1^): 3418, 1696 (C=O), 1304, 1253, 1038, 785, 657. ^1^H- NMR (DMSO-d_6_, *δ*, ppm): 2.02 (3H, s, CH_3_); 6.33 (1H; d, J = 2.24 Hz; H-6 of catechol ring); 6.50 (1H; d, J = 2.24 Hz; H-2 of catechol ring); 7.1 (2H; d, J = 6.9 Hz; H-2 and H-6 of phenyl ring); 7.31 ( 3H, m, H-3, H-4 and H-5 of phenyl ring); 8.09 (4H, m, Hs of indandione ring); 8.37 (1H, s, OH); 9.30 (1H, s, OH). ^13^C-NMR (DMSO, *δ*, ppm): 16 (CH_3_), 66.7 (C-2 of indandione ring), 113.2, 120.7, 123.9, 124.5, 127.1, 127.5, 128.4, 128.5, 137.1, 138.1, 140.7, 143.1, 144.8, 199.5 (C=O). ESI-MS: Observed (M+H)^+^ = 345, (M+Na)^+^ = 367 (Calcd for C_22_H_16_O_4_ = 344.36). *Anal. *Calcd for C_22_H_16_O_4_: C, 76.73; H, 4.68. Found: C, 76.8; H, 4.68.

*2-(4-Fluorophenyl)-2-(3,4-dihydroxyphenyl)-2H-indene-1,3-dione (16a)*

Yield: 41%, mp: 196-199 ^°^C. IR (KBr) cm^-1^: 3435, 1695 (C=O). ^1^H-NMR (DMSO-d_6_, *δ*, ppm): 6.39 (1H; dd, J = 8.3 Hz, J = 2.35 Hz; H-6 of catechol ring); 6.58 (1H; d, J = 2.35 Hz; H-2 of catechol ring); 6.67 (1H; d, J = 8.2 Hz; H-5 of catechol ring); 7.16 ( 4H, m, Hs of fluorophenyl ring); 8.1 (4H, m, Hs of indandione ring); 9.2 (2H, bs, OHs). ^13^C-NMR (DMSO, *δ*, ppm): 66.5 (C-2 of indandione ring), 115.6, 115.9, 116.1, 119.5, 124.5, 128.4, 131.1, 131.3, 134.7, 134.8, 137.6, 141.2, 145.8, 146.0, 159.9, 163.8, 199.7 (C=O). ESI-MS: Observed (M+H)^+^ = 349, (M+Na)^+^ = 371 (Calcd for C_21_H_13_FO_4_, = 348.32). *Anal. *Calcd for C_21_H_13_FO_4_: C, 72.41; H, 3.76. Found: C, 72.15; H, 3.76

*2-(4-Fluorophenyl)-2-(3,4-dihydroxy-5-methylphenyl)-2H-indene-1,3-dione (16b)*

Yield: 31%, mp: 174-177 ^°^C. IR (KBr) cm^-1^: 3433, 1690 (C=O). ^1^H-NMR (DMSO-d_6_, *δ*, ppm): 2.01 (3H, s, CH_3_); 6.3 (1H; d, J = 2.3 Hz; H-6 of catechol ring); 6.48 (1H; d, J = 2.3 Hz; H-2 of catechol ring); 7.15 (4H, m, Hs of fluorophenyl ring); 8.09 (4H, m, Hs of indandione ring); 8.40 (1H, s, OH); 9.32 (1H, s, OH). ^13^C-NMR (DMSO, δ, ppm): 16 (CH_3_), 66.0 (C-2 of indandione ring), 113, 115.1, 115.4, 120.6, 124.0, 124.6, 127.1, 130.6, 130.7, 134.1, 134.2, 137.2, 140.6, 143.2, 144.8, 159.4, 163.2, 199.3 (C=O). ESI-MS: Observed (M+H)^+^ = 363, (M+Na)^+^ = 385 (Calcd for C_22_H_15_FO_4_ = 362.35). *Anal*. Calcd for C_22_H_15_FO_4_: C, 72.92; H, 4.17. Found: C, 72.7; H, 4.17.

*2-(4-Chlorophenyl)-2-(3,4-dihydroxyphenyl)-2H-indene-1,3-dione (17a)*

Yield: 27%, mp: 207-209 ^°^C. IR (KBr) cm^-1^: 3417, 1686 (C=O). ^1^H-NMR (DMSO-d_6_, *δ*, ppm): 6.39 (1H; dd, J = 8.3 Hz, J = 2.3 Hz; H-6 of catechol ring); 6.58 (1H; d, J = 2.3 Hz; H-2 of catechol ring); 6.67 (1H; d, J = 8.3 Hz; H-5 of catechol ring); 7.13 (2H; d, J = 8.7 Hz; H-2 and H-6 of chlorophenyl ring); 7.40 (2H; d, J = 8.7 Hz; H-3 and H-5 of chlorophenyl ring); 8.1 (4H, m, Hs of indandione ring); 9.07 (1H, bs, OH); 9.10 (1H, bs, OH). ^13^C-NMR (DMSO, δ, ppm): 66.6 (C-2 of indandione ring), 116.0, 116.5, 119.6, 124.5, 128.1, 128.9, 131.0, 132.9, 137.6, 137.7, 141.1, 145.9, 146.0, 199.5 (C=O). ESI-MS: Observed (M+H)^+^ = 365, (M+Na)^+^ = 387 (Calcd for C_21_H_13_ClO_4 _= 364.78). *Anal*. Calcd for C_21_H_13_ClO_4_: C, 69.14; H, 3.59. Found: C, 69.24; H, 3.59.

*2-(4-Chlorophenyl)-2-(3,4-dihydroxy-5-methylphenyl)-1H-indene-1,3-dione (17b)*

Yield: 8%, mp: 170-173 ^°^C. IR (KBr) cm^-1^: 3417, 1690 (C=O). ^1^H-NMR (DMSO-d_6_, *δ*, ppm): 2.01 (3H, s, CH_3_); 6.32 (1H; d, J = 2.22 Hz; H-6 of catechol ring); 6.48 (1H; d, J = 2.2 Hz; H-2 of catechol ring); 7.13 (2H; d, J = 8.6 Hz; H-2 and H-6 of chlorophenyl ring); 7.40 (2H; d, J = 8.6 Hz; H-3 and H-5 of chlorophenyl ring); 8.1 (4H, m, Hs of indandione ring); 8.4 (1H, bs, OH); 9.30 (1H, bs, OH). ^13^C-NMR (DMSO, *δ*, ppm): 16.5 (CH_3_), 66.6 (C-2 of indandione ring), 116, 121, 124.5, 125, 128.9, 131.3, 133, 137.7, 141.2, 143 146, 151, 155, 199 (C=O). ESI-MS: Observed (M+H)^+^ = 379, (M+Na)^+^ = 401 (Calcd for C_22_H_15_ClO_4_ = 378.81). *Anal. *Calcd for C_22_H_15_ClO_4_: C, 69.75; H, 3.99. Found: C, 69.8; H, 3.99.

*2-(4-Bromophenyl)-2-(3,4-dihydroxyphenyl)-2H-indene-1,3-dione (18a)*

Yield: 16%, mp: 204-205 ^°^C. IR (KBr) cm^-1^: 3325, 1691 (C=O), 1248, 791, 713, 652. ^1^H-NMR (DMSO-d_6_, δ, ppm): 6.39 (1H; dd, J = 8.3 Hz, J = 2.3 Hz; H-6 of catechol ring); 6.58 (1H; d, J = 2.3 Hz; H-2 of catechol ring); 6.67 (1H; d, J = 8.3 Hz; H-5 of catechol ring); 7.07 (2H; d, J = 8.6 Hz; H-2 and H-6 of bromophenyl ring); 7.54 (2H; d, J = 8.6 Hz; H-3 and H-5 of bromophenyl ring); 8.1 (4H, m, Hs of indandione ring); 9.08 (2H, bs, OHs). ^13^C-NMR (DMSO, *δ*, ppm): 66.2 (C-2 of indandione ring), 115.5, 115.6, 119.0, 121.0, 124.0, 127.5, 130.8, 131.3, 137.2, 137.5, 140.6, 145.4, 145.5, 198.8 (C=O). ESI-MS: Observed (M+H)^+^ = 409,411, (M+Na)^+^ = 431,433 (Calcd for C_21_H_13_BrO_4_ = 409.23). *Anal. *Calcd for C_21_H_13_BrO_4_: C, 61.63; H, 3.20. Found: C, 61.7; H, 3.20.

*2-(4-Bromophenyl)-2-(3,4-dihydroxy-5-methylphenyl)-2H-indene-1,3-dione (18b)*

Yield: 14%, mp: 194-195 ^°^C. IR (KBr) cm^-1^: 3497, 3440, 1685 (C=O), 1252, 817, 660. ^1^H-NMR (DMSO-d_6_, *δ*, ppm): 2.01 (3H, s, CH_3_); 6.32 (1H; d, J = 2.2 Hz; H-6 of catechol ring); 6.48 (1H; d, J = 2.2 Hz; H-2 of catechol ring); 7.06 (2H; d, J = 8.6 Hz; H-2 and H-6 of bromophenyl ring); 7.54 (2H; d, J = 8.6 Hz; H-3 and H-5 of bromophenyl ring); 8.1 (4H, m, Hs of indandione ring); 8.4 (1H, bs, OH); 9.4 (1H, bs, OH). ^13^C-NMR (DMSO, δ, ppm): 16.5 (CH_3_), 66.8 (C-2 of indandione ring), 113.5, 121.1, 121.5, 124.5, 125.1, 127.2, 131.4, 131.8, 137.7, 138.1, 141.1, 143.8, 145.4, 199.4 (C=O). ESI-MS: Observed (M+H)^+^ = 423,425, (M+Na)^+^ = 445,447 (Calcd for C_22_H_15_BrO_4_ = 423.26). *Anal. *Calcd for C_22_H_15_BrO_4_ : C, 62.43; H, 3.57. Found: C, 62.45; H, 3.57. *2-(3,4-dihydroxyphenyl)-2(4-methoxyphenyl)-2H-indene-1,3-dione (19a)*

Yield 27%, mp 160-164 ^°^C. IR (KBr) cm^-1^: 3257, 1708, 1676, 1583, 1518, 1254, 1230, 818, 769.^ 1^H-NMR (CDCl_3_ -d_6_, *δ*, ppm): 3.63 (3H, s, OCH_3_); 6.45 (1H; dd, J = 8.3 Hz, J = 1.8 Hz; H-6 of catechol ring); 6.62 (1H; d, J = 8.3 Hz; H-5 of catechol ring); 6.65 (1H; d, J = 1.8 Hz; H-2 of catechol ring); 6.69 (2H; d, J = 8.7 Hz; H-3 and H-5 of methoxyphenyl ring); 7.05 (2H; d, J = 8.7 Hz; H-2 and H-6 of methoxyphenyl ring); 7.77 (2H, m, H-5 and H-6 of indandione ring); 7.92 (2H, m, H-4 and H-7 of indandione ring); 7.7 (1H, bs, OH); 7.9 (1H, bs, OH). ^13^C-NMR (DMSO, *δ*, ppm): 55 (OCH_3_), 66.0 (C-2 of indandione ring), 113.8, 115.4, 115.8, 119.1, 123.9, 128.3, 129.6, 129.9, 137.0, 140.7, 145.1, 145.3, 158.5, 199.7 (C=O). ESI-MS: Observed (M+H)^+^ = 361, (M+Na)^+^ = 383 (Calcd for C_22_H_16_O_5 _= 360.36). *Anal. *Calcd for C_22_H_16_O_5_: C, 73.33; H, 4.48. Found: C, 73.30; H, 4.48.

*2-(3,4-dihydroxy-5-methylphenyl)-2-(4-methoxyphenyl)-2H-indene-1,3-dione (19b)*

Yield: 42%, mp: 141-142 ^°^C. IR (KBr) cm^-1^: 3413, 1687 (C=O), 1513, 1251, 1035, 658. ^1^H-NMR (DMSO-d_6_, *δ*, ppm): 2.01 (3H, s, CH_3_); 3.73 (3H, s, OCH_3_); 6.30 (1H, s, H-6 of catechol ring); 6.47 (1H; s, J = 2.2 Hz; H-2 of catechol ring); 6.9 (2H; d, J = 8.0 Hz; H-3 and H-5 of methoxyphenyl ring); 7.05 (2H; d, J = 8.0 Hz; H-2 and H-6 of methoxyphenyl ring); 8.1 (4H, m, Hs of indandione ring); 8.5 (1H, bs, OH); 9.4 (1H, bs, OH). ^13^C-NMR (DMSO, *δ*, ppm): 16.5 (CH_3_), 55.5 (OCH_3_), 66.6 (C-2 of indandione ring), 113.7, 114.3, 121.2, 124.4, 124.9, 128.1, 130.2, 130.3, 137.5, 141.2, 143.2, 145.2, 159.0, 200.3 (C=O). ESI-MS: Observed (M+H)^+^ = 375, (M+Na)^+^ = 397 (Calcd for C_23_H_18_O_5_ = 374.39). *Anal. *Calcd for C_23_H_18_O_5_: C, 73.79; H, 4.85. Found: C, 73.69; H, 4.84. 

*Platelet aggregation studies*

Blood was obtained from healthy volunteers who did not take any medication for 14 days and were fasting over night prior to the study. Platelet rich plasma (PRP) was prepared by the centrifugation of citrated blood at 100g for 10 min. The residual blood was centrifuged at a speed of 1500 g for 15 min to give platelet poor plasma (PPP). Platelets were counted under microscope and the platelet count was adjusted to (250 ± 25)×10^9^/L. Aliquots of 200 μL of PRP were distributed in the test cuvettes and placed in incubation chamber of APACT-4004 aggregometer (LABiTec, Ahrensburg, Germany), at 37°C. Platelet aggregation was measured using PRP after activation by the addition of ADP or AA according to Born method (19). The test compounds were dissolved in DMSO (at 0.05% final concentration) and added to the PRP, 5 min prior to the activation with ADP or AA. The extent of aggregation was quantified by determining the maximum height of the curve. The platelet aggregation inhibitory activity was expressed as percent inhibition by comparison with that measured for the vehicle (DMSO) alone.

## Results and Discussion

**Figure 6:**
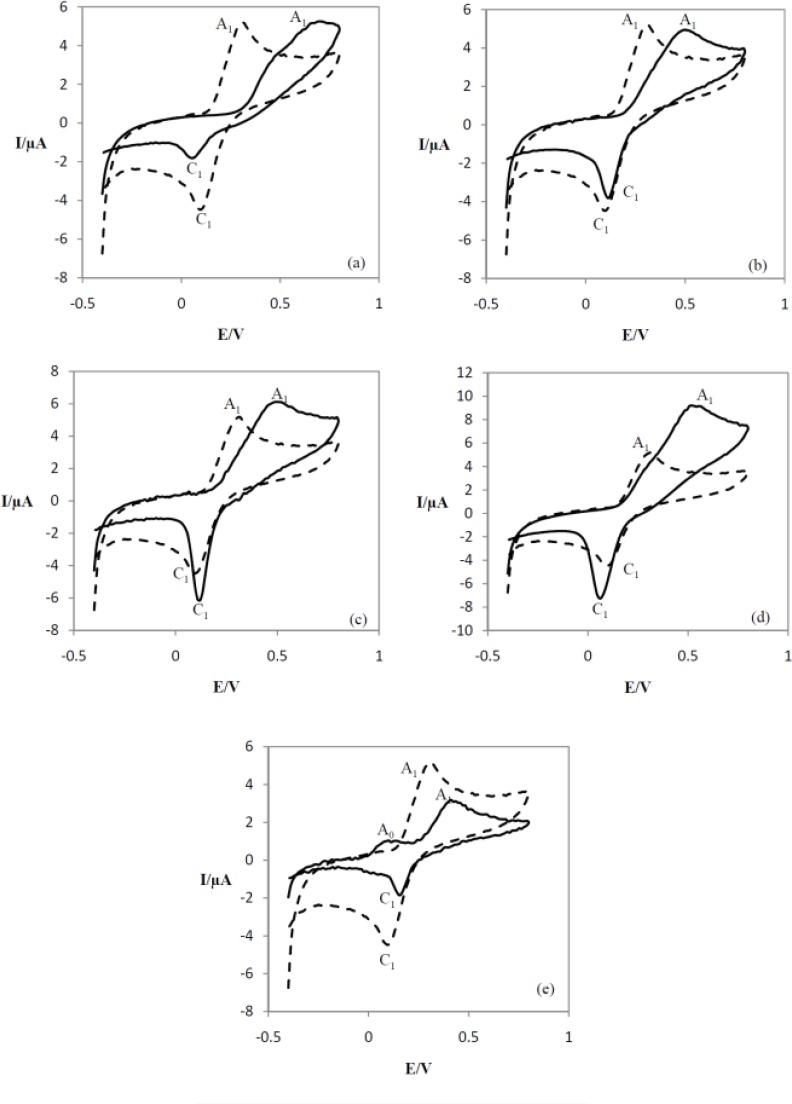
Cyclic voltammograms of **12 **in absence of any indandione derivatives (compounds **7-11**) (dashed line) and cyclic.voltammograms of **12 **in presence of (a) **7**, (b) **8**, (c) **9, **(d) **10 **and (e) **11 **(solid lines), V = 150 mVs^-1^

**Figure 7:**
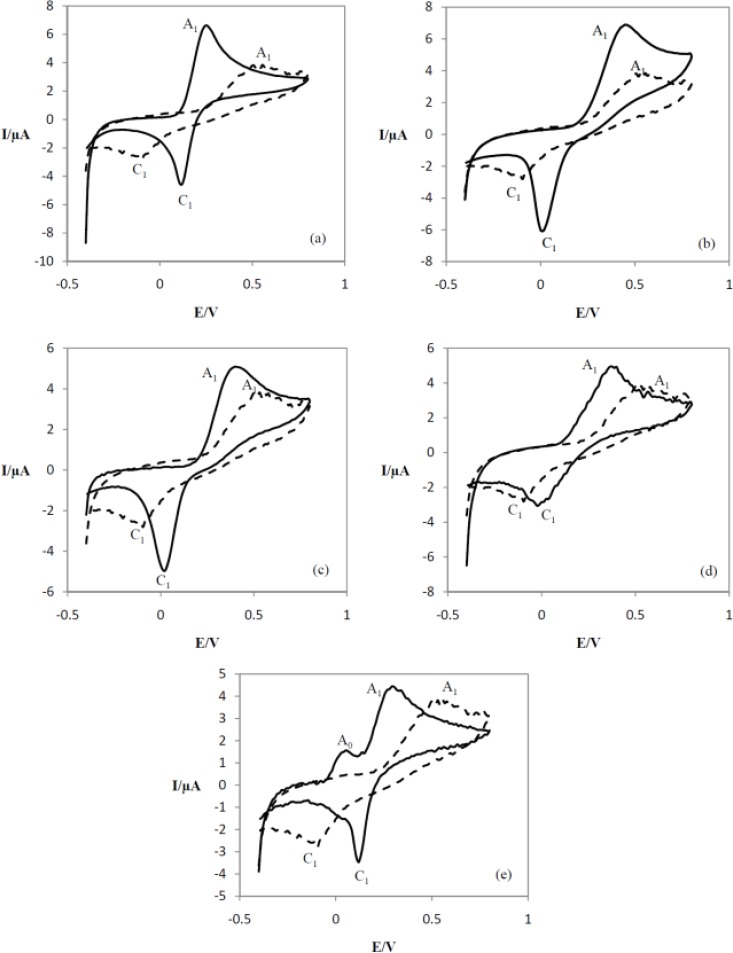
Cyclic voltammograms of **13 **in absence of any indandione derivatives (compounds **7-11**) (dashed line) and cyclic voltammograms of catechol in presence of (a) **7**, (b) **8**, (c) **9**, (d) **10 **and (e) **11 **(solid lines), V = 150 mVs^-1^.

The electrochemical behavior of catechol (12) and 3-methylcatechol (13) were studied in the absence and presence of the nucleophiles 7-11 (Figure 6 and 7).

Voltammograms of 12 and 13 show anodic peaks at 0.3 and 0.53 V and show cathodic peaks at 0.1 and **-**0.1 V, respectively. Anodic peaks indicate the oxidation of 12 and 13 to their corresponding *o*-benzoquinones which are reduced back to their initial catechol form by reversing the voltage. The ratio of the oxidation and reduction current amplitudes 12 and 13 in the oxidation and reduction process were equal to unity. This phenomenon shows the stabilization of *o*-benzoquinone produced under surface of electrodes. Side reactions, such as dimerization or hydroxylation, (16, 20) are too slow to be observed on the time scale of cyclic voltammetry. As shown in Figure 6, when compounds 7-10 were separately added to the system containing catechol, the anodic peak (A_1_) shifted positively in all cases and it was decreased for the mixture of 12 and 11. The cathodic peak (C_1_) current for 12 decreased in the presence of 7, 8 and 11 while it increased in the presence of 9 and 10. The positive shift of anodic potential can be related to oxidation of 12 and its corresponding products, or it could be due to the formation of a thin film on the surface of the electrodes (16). As it appears from Figure 7, the anodic peak (A_1_) of 13 increased and shifted negatively in the presence of 7-11. A new anodic peak (A_0_) appeared in the presence of 11. This new peak is related to electrochemical oxidation of intermediate.

The results show that 12 and 13 are oxidized to their corresponding *o*-quinones. The nucleophiles then attack the *o*-quinones and new dihydroxybenzene derivatives are formed.

Based on the spectroscopic data obtained for the products, it seems that the nucleophiles have attached to the *o*-quinones through a 1,4-Michael addition.

In the case of 13, the *o*-quinone which is formed from this compound, could be attacked by the nucleophiles through a 1,4- Michael addition at two different positions, leading to two different products but the characterization of the products show that, due to the steric effects, the nucleophiles have attacked the sterically less hindered carbon, and only one major product has been obtained. The^ 1^H-NMR spectra of compounds 15b-19b show two peaks in aromatic region with meta splitting pattern confirming the overall reaction mechanism for anodic oxidation of 12 and 13 in the presence of 1,3-indandione derivatives 7-11**, **which is presented in Figure 4.

In case of reaction between 3,4-dihydroxybenzoic acid (14) and the nucleophiles 7-11, the desired products were not obtained. This could be due to the incompatibility of the carboxylic acid functional group and the carbanion which is supposed to be formed at position 2 of indandione ring.

The results of *in-vitro *anti-platelet activity were summarized in Table 1 and 2. 

**Table 1:** Effect of 2-aryl-1,3-indandione derivatives at 0.5 mM concentration on *in-vitro *platelet aggregation induced by ADP.

**Compound**	**ADP (5 μM)**	
	**Aggregation** ^a^ **(%)**	**Inhibition (%)**
Control	77.196 ± 5.49	
Indomethacin^b^	70.93 ± 4.53	8.11
ASA^b^	61.1 ± 1.23	21
Quercetin	75.6 ± 2.26	2
7	51.42 ± 4.6	33.39
15a	50.28 ± 1.30	34.86
15b	48.21 ± 1.58	37.54
8	49.03 ± 3.76	36.48
16a	47.10 ± 0.87	38.98
16b	44.9 ± 2.58	41.83
9	47.7 ± 3.46	38.20
17a	49.1 ± 5.05	36.39
17b	ND^c^	ND
10	44.01 ± 2.55	42.98
18a	51.18 ± 1.75	33.70
18b	48.38 ± 6.64	37.32
11	57.75 ± 4.26	25.19
19a	45.12 ± 2.69	41.55
19b	53.28 ± 3.01	30.98

**Table 2:** Inhibitory effect of 2-aryl-1,3-indandione derivatives on *in-vitro *platelet aggregation induced by arachidonic acid (AA).

**Compound**	**AA (1.35 mM)**		
	**500 (μM)**	**250 (μM)**	**IC**_50_ ** (μM)**
ASA^a^	95%	95%	0.24
Quercetin	36%	1%	353
7	0%	0%	ND^b^
15a	100%	100%	196
15b	100%	100%	136
8	0%	0%	ND
16a	100%	3%	392
16b	100%	8%	411
9	5%	0%	ND
17a	16%	16%	ND
17b	ND	ND	ND
10	0%	0%	ND
18a	100%	12%	400
18b	100%	15%	251
11	0%	0%	ND
19a	100%	50%	243
19b	100%	10%	429

^a ^ASA (Acetylsalicylic acid) was used as a positive control. ^b ^ND = not determined

Interestingly, all the synthesized 2,2-diaryl-1,3-indandione derivatives (15a-b, 16a-b, 17a-b, 18a-b, 19a-b) exhibited 100% inhibition of platelet aggregation at 500 μM when AA was used as agonist and these compounds exhibited no significant inhibitory activity against ADP induced platelet aggregation.

**Figure 8:**
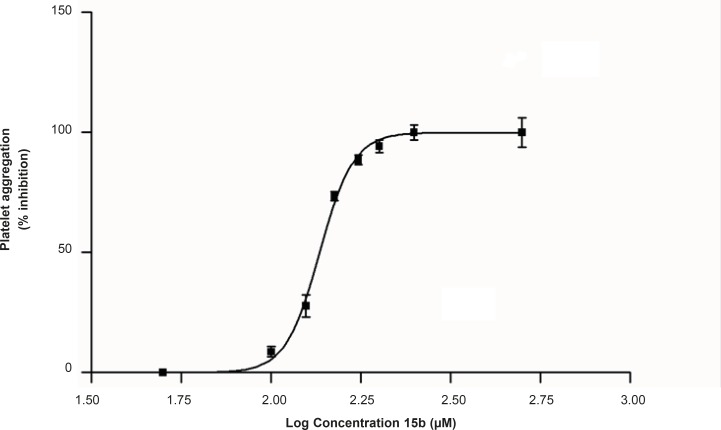
Dose-response curve for compound 15b. Arachidonic acid (1.35 mM) used as platelet aggregation inducer and data shown as mean ± SE (n = 3).

Comparing the results obtained for the new series (15a-b, 16a-b, 17a-b, 18a-b, 19a-b), with those obtained for their corresponding parent compounds (7-11), reveals the following facts: In the ADP-induced aggregation studies, no statistically significant difference was observed between the activities of parent indandiones (7-11) and catechol and 3-methylcatechol series (15a-19a and 15b**-**19b). In fact, taking the high concentration of tested compounds (500 μM) into account, the compounds could be considered completely inactive towards ADP-induced platelet aggregation. On the other hand, comparison of the effects of the same series of compounds on the platelet aggregation induced by arachidonic acid (AA) shows another pattern: all the compounds 7-11, caused no inhibition on platelet aggregation induced by AA. On the contrary, all 15a-19a and 15b**-**19b compounds showed satisfactory platelet inhibitory activity at 500 μM. It appears that incorporation of catechol or methylcatechol ring to the structure of compounds 7-11 has caused the emergence of antiplatelet activity in these compounds. In particular compounds 15a, 15b and 19a still showed a noticeable antiplatelet activity even at lower concentrations. These findings are in agreement with the previously reported studies which indicate that quercetin inhibits platelet aggregation induced by AA and is less active against platelet aggregation induced by ADP (21-22). The calculated IC_50 _values for the most active compounds indicated that the new indandione derivatives 15a, 15b, 18b and 19a are more potent than quercetin. The dose-response curve for compound 15b is presented in Figure 8.

It could be therefore concluded that indandione derivative with catechol ring on their position 2, are more effective in interfering with the cyclooxygenase (COX)-TXA_2_ syntheses pathway than inhibiting the ADP receptor. In other words, it is highly speculated that they exert their effects with an ASA-like profile (21-23).

On the basis of the results obtained in the present study the most promising compounds (15a, 15b and 19a) are currently under further pharmacological investigation in order to assess their potential antithrombotic activity *in-vivo*. Moreover, complementary detailed studies will be performed to identify their molecular targets.

## Conclusion

In summary, we have synthesized new indandione derivatives (15a-19a and 15b-19b) by electrosynthesis method and evaluated their antiplatelet aggregation activity against ADP and AA as the aggregation inducers. Compounds 15a, 15b and 19a showed significant antiplatelet aggregation when arachidonic acid was used as the inducer. These compounds could be considered as both antiplatelet and anticoagulant agents and further investigations are in progress to prove and optimize their dual acting.
